# Current Hospital Topics

**Published:** 1907-01-19

**Authors:** 


### HOSPITAL ADMINISTRATION.

### CONSTRUCTION AND ECONOMICS.

### CURRENT HOSPITAL TOPICS.

#### Salford Royal Hospital.

At the December meeting of the Board of Management of this hospital the resignation of the chairmanship, through ill health, was received from Mr. A. Langley Dickins. Mr. Dickins was appointed a member of the Board during the time he was Mayor of Salford, in 1889, and became Chairman in 1898. A resolution was unanimously adopted recording the gratitude of the Governors for the active part Mr. Dickins has taken in the administration of the hospital for so many years, and especially for the valuable services he has rendered as its Chairman.

#### Charing Cross Hospital.

The vacant secretaryship of Charing Cross Hospital has at length been filled by the appointment of Mr. Walter Alvey, who has been in the service of the Hospital for the past ten years, first as clerk, and then as assistant secretary and steward. Mr. Alvey has been carrying on the secretarial work of the hospital since Mr. Reade's retirement on pension in June last, and has exhibited marked ability, energy, and enterprise. The appointment is a most popular one among Mr. Alvey's co-workers in the hospital world, where he is well known and deservedly respected.

#### The Gratitude of Hospital Patients.

Speaking at the recent festival dinner of the Royal Southern Hospital, Liverpool, the President, Mr. W. Adamson, stated that it was almost a lifetime since he joined the committee, in 1864, and so first took an active part in the management of the hospital. Mr. Adamson gave some interesting particulars of the appreciation by patients of the efforts made on their behalf. In one case a patient wrote: "I make this offer of myself voluntarily, and perfectly free from compulsion, being a healthy and well developed man, free from any hereditary mental or physical taint, aged 22, as a subject for any surgical operation, major or minor, or medical treatment." Mr. Adamson also stated that recently the Medical Board granted permission to the patients to smoke between the hours of seven and eight in the morning, as well as in the evening. After the new regulations came into force, one of the committee, when going his rounds, was received by the patients with three cheers, followed by, "For he's a jolly good fellow."

#### Example Better than Precept.

The example of the late George Herring, and the form his munificence took in regard to the Hospital Sunday Fund, have stimulated the citizens of Liverpool to follow his methods. The first Hospital Sunday collections in Liverpool in 1871 realised £4,740, and last year the Sunday and Saturday collections had increased to £15,000. A general wish is now expressed in Liverpool that this amount should be doubled without delay. Having this in view, the Lord Mayor announces that ten citizens, prominent in philanthropic efforts, have come forward with the generous offer to each guarantee 10 per cent. on the amount collected this year, over and above the £15,000 contributed last year. If, therefore, the citizens of Liverpool bestir themselves, there should be little difficulty in increasing the collections, at once, from £15,000 to £22,500, when the guarantors would make up the total collection for 1907 to £30,000. We hope this result may be achieved, and we rejoice to know that the spirit of Mr. Herring's munificence and example is extending to the provinces.

#### Bradford Royal Infirmary.

The vacancy caused in the office of secretary to this institution by the appointment of Mr. Edmund Forster to the Derby Infirmary, has been filled by the election of Mr. John J. Barron, assistant secretary of the Victoria Infirmary, Glasgow. The Committee of Selection carefully sifted seventy applications, first rejecting those candidates who had had no previous experience of hospital work, and then reducing the remainder to seven, and ultimately to three, from whom Mr. Barron was elected by the governors. Mr. Barron was born in Scotland of English parentage, is thirty-three years of age, and has been thoroughly trained in all the duties of the secretarial department of a general hospital. Mr. Barron has worked for fourteen years as assistant-secretary at the Victoria Infirmary, Glasgow, where his services have been much appreciated, and his testimonials include excellent testimony from the Board of Management and the principal members of the medical staff. Mr. Barron is to commence work in Bradford next month, and we wish him a long and prosperous career in his new post.

**The Royal Infirmary, Edinburgh.**

THE report of the managers of this institution for the year ending September 30, 1906, is full of interest. It illustrates the developments which modern science has imposed upon the authorities of one of the oldest and most efficient hospitals in the country, and gives some insight into the growth and expenditure now entailed upon an institution of this kind. A new surgical out-patient and accident department has been established. This consists of four pairs of dressing-rooms, each containing a separate room for men and for women; one is for fracture cases, one for aseptic cases, one for septic cases, and one for the use of women students. There is a waiting-hall facing the new entrance, which is connected by a corridor with an operating and lecture theatre, with accommodation for about 100 students. Near by are three casualty wards, with four beds each, for the observation of serious cases. The new department contains accommodation for the resident house surgeon, the sister in charge, and a workroom for the assistant surgeon, who is responsible for the working of the whole department. The assistant-surgeons take charge of the department in rotation, for four months at a time. This new department, with a staff of three resident surgeons and seven nurses, has entailed a net expenditure of about £700 a year. The new medical electrical department, and the new bath department, fitted with the latest improvements and appliances, have added to the expenditure. An increase to the nursing and serving staff has cost £300, in addition to a large outlay owing to the substitution of paid for unpaid nurses under the longer period of training; massage treatment, lately introduced, has cost £200, and the telephonic staff £130. Further additions to expenditure have been due to the reorganisation and extension of the pathological, fire, and works departments, and to numerous minor improvements and additions, together involving an increased expenditure of over £5,000 per annum, or more than £6 per occupied bed. Economies have, however, been introduced to such an extent that, this increase of £6 per occupied bed has been reduced, to a net increase of 7s. 11½d. per bed for the year, as compared with the expenditure in 1899. These results reflect great credit upon the managers and their officers, credit which is increased by the knowledge that although the expenditure in 1906 (£52,116) was £2,000 more than in the previous year, the total revenue, including legacies and donations, has exceeded the total expenditure in 1906 by £3,000. A study of the accounts for previous years shows, that, the authorities in Edinburgh are careful financiers, who decline to incur expenditure before they can see their way to secure the funds to meet it. They have recently decided to appoint a new officer as organising secretary, whose whole time will be devoted to the stimulation of interest in the Royal Infirmary, and to the organisation of collections on its behalf in Edinburgh and throughout the country, particularly from those classes who benefit directly from the institution. The wise economy and sound financial system, in force at this institution, have been maintained without any diminution in the progressive development of its buildings or its work. We commend this fact to the consideration of all hospital managers everywhere.

